# The Content of Bioactive Compounds and Technological Properties of Matcha Green Tea and Its Application in the Design of Functional Beverages

**DOI:** 10.3390/molecules28207018

**Published:** 2023-10-10

**Authors:** Katarzyna Najman, Anna Sadowska, Monika Wolińska, Katarzyna Starczewska, Krzysztof Buczak

**Affiliations:** 1 Department of Functional and Organic Food, Institute of Human Nutrition Sciences, Warsaw University of Life Sciences, Nowoursynowska 159c, 02-776 Warsaw, Poland; katarzyna_najman@sggw.edu.pl (K.N.);; 2 Department of Surgery, Faculty of Veterinary Medicine, Wroclaw University of Environmental and Life Science, Pl. Grunwadzki 51, 50-366 Wroclaw, Poland; krzysztof.buczak@upwr.edu.pl

**Keywords:** Matcha, *Camelia sinensis*, green tea, polyphenols, antioxidant activity, L*a*b*, WHC, WSI, °Brix, pH, osmolality, functional drinks

## Abstract

Matcha is a powdered green tea obtained from the *Camellia sinensis* L. plant intended for both “hot” and “cold” consumption. It is a rich source of bioactive ingredients, thanks to which it has strong antioxidant properties. In this research, an organoleptic evaluation was carried out, and the physical characteristics (i.e., instrumental color measurement (L*a*b*), water activity, water solubility index (WSI), water holding capacity (WHC) of 10 powdered Matcha green teas, and in the 2.5% Matcha water solutions, pH, °Brix and osmolality were tested. Also, the content of phenolic ingredients, i.e., selected phenolic acids, flavonoids and total polyphenols, was assessed. The content of chlorophyll, vitamin C and antioxidant potential were also examined. Matcha M-4 was used to design two functional model beverages, in the form of ready-to-use powdered drinks, consisting of Matcha green tea, protein preparations, inulin, maltodextrin and sugar. The obtained powdered drink, when dissolved in the preferred liquid (water, milk, juice), is regenerative, high-protein and rich in bioactive ingredients from the Matcha drink, with prebiotic properties derived from the added inulin. The beverage is also characterized by low osmolality. It can be recommended as a regenerating beverage for a wide group of consumers, athletes and people with deficiencies, among others protein, and elderly people, as well as in the prevention and supportive treatment of bone and joint tissue diseases.

## 1. Introduction

In recent years, there has been an increasing interest in functional products and low-processed food naturally rich in antioxidants that can protect against the harmful effects of free radicals and oxidative stress. Such products, rich in bioactive substances, include Matcha green tea, which is gaining more and more popularity around the world [1,2].

Matcha is obtained from the *Camellia sinensis* L. plant (*Thea sinensis*), which in the botanical classification belongs to the plant kingdom (*Plantae*), the vascular group (*Tracheophyta*), the tea family (*Theaceae*), the genus (*Camellia*). There are two types of Matcha tea: Matcha-Koicha and Matcha-Usucha, intended for making a decoction or infusion, both “hot” and “cold”. Koicha has the form of thick, viscous tea with an intense, bitter aroma, while Usucha has a diluted form due to the fact that it is brewed in more water than Matcha-Usucha [3,4,5].

Matcha comes from the regions of Nishio and Uji Tawara in Aichi Prefecture (Japan). Its history dates back to the 13th century when Japanese Zen monks used it as a means of relaxing and maintaining concentration during long hours of meditation. It is commonly consumed during the Japanese Cha-no-you tea ceremony. It is part of the Japanese culinary culture and is gaining more and more popularity among consumers of other cultures and regions of the world [1,2,3,6].

Three weeks before harvest, tea bushes are covered with reed mats to protect the young leaves from direct sunlight [7]. Thanks to this, the content of amino acids and bioactive compounds (especially chlorophyll and theanine) increases; this is responsible for the characteristic color and taste of Matcha [1]. The tea harvest begins at the end of April; the first one lasts until the end of May, the next ones fall at the turn of June and July, while the third harvest falls in August. The leaves collected from the top of the shoot are of the best quality [6,8]. 

Black and Oolong teas are produced as a result of total or partial fermentation of *Camelia sinensis* L. leaves, leading to their typical black or brown color, which is a result of enzymatic oxidation of catechins present in leaves [9,10]. Unlike them, green tea (from which Matcha is made) is produced without the fermentation process [8,11]. After the leaves are harvested, the first stage in the production of green teas is high-temperature heat treatment (steaming, roasting or steaming at 80–90 °C), preventing oxidation [12,13]. At this stage, enzymes (polyphenol oxidase and peroxidase), catalyzing the reactions of catechins contained in the leaves with oxygen, are inactivated [9,10,14]. This prevents fermentation processes and inhibits the decomposition of color pigments contained inside the leaves, which allows the tea to retain its intense green color [11,14]. The next step is drying at a moderate temperature, thanks to which the leaves shrink, and finally grinding them into a powder in granite mills, i.e., slowly rotating stones [8,12]. The final stage is roasting at a high temperature, which allows for an intense tea flavor [8,12,13]. 

Due to the unique composition of bioactive compounds, Matcha has a wide range of health benefits. It is characterized by a high content of antioxidant compounds, which results from the method of shade cultivation and leads to the production of larger amounts of amino acids and bioactive compounds [1]. Matcha contains high concentrations of phenolic acids], rutin, quercetin [2,13,15,16,17,18,19,20,21,22], theanine, chlorophyll and other carotenoid [12,23] amounts exceeding traditional green teas. Due to the richness of bioactive ingredients, especially those with antioxidant properties (polyphenols, mainly flavonoids) [12,15,24,25,26,27], it is more and more often used in the prevention and treatment of many civilization diseases, e.g., heart disease, diabetes and hypertension [1,13,22,24,28,29]. Its infusions can also be used in the prevention of inflammatory [1,30,31] or viral diseases [20,32]. Matcha may have a positive effect on weight loss by reducing the level of triglycerides, fat and glucose in the blood and additionally may contribute to increasing muscle mass [22,28,29]. Matcha is also known for its anti-aging properties [33].

In recent years, Matcha has become an increasingly popular product among consumers [6]. On the food market, Matcha is most often found in powdered form [16,24,26], intended for both “hot” and “cold” consumption [4,34], but it is also increasingly used as an additive to various products, such as bars, jellies, cakes, cookies, chocolates, candies, puddings, drinks, cocktails or ice cream [4,34], becoming a promising ingredient in the functional food industry [22,35,36,37].

Despite the ever-growing popularity of this product, knowledge about Matcha green tea is insufficient and constantly expanded with scientific research, which is why this research was carried out in two stages. In the first stage of this research, the physicochemical and bioactive properties of 10 types of Matcha green tea products available on the Polish market were assessed. Physicochemical tests (solubility, degree of water binding, color, osmolality, soluble solids content) were carried out in powdered teas. The content of bioactive components, i.e., selected phenolic acids and flavonoids, total polyphenols, chlorophyll, vitamin C and antioxidant activity, were also assessed. In the second stage of the research, the composition of powdered beverages containing Matcha green tea, selected from among those tested in the first stage of the research, was designed as a source of bioactive substances as well as to shape the taste profile of the designed beverages. Therefore, in this study, research was conducted on designing innovative functional beverages in powder form, containing bioactive ingredients from Matcha and easily digestible proteins from milk or enzymatically hydrolyzed collagen proteins and additionally containing a prebiotic (inulin). Such composition and convenient form (powder) allow direct consumption of the drink after dissolving it in water, milk or juice according to consumer preference. Designed functional beverages can be intended for a wide group of consumers, especially physically active people, as a recovery drink after exercise due to its rich composition and low osmolality (hypotonic and isotonic drink), as well as for the elderly in the prevention and treatment of diseases of joint and bone tissue.

## 2. Results

### 2.1. Physicochemical and Bioactive Properties of Matcha Green Tea

#### 2.1.1. Physicochemical Properties of Matcha Green Tea 

##### Organoleptic Evaluation of Matcha Green Tea Powders

**Figure 1:**
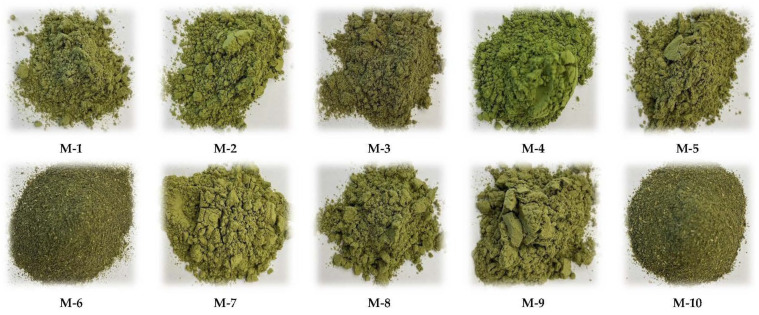
The general appearance of the tested Matcha green tea powders. Symbols from M-1 to M-10 indicate the 10 tested types of green Matcha tea.

Figure 1 presents the general appearance of the tested samples of 10 market products—Matcha green tea powders. 

As results from the organoleptic evaluation of Matcha teas, the tested samples were characterized by a diversified general appearance and color. Among the evaluated products, samples M-6 and M-10 were characterized by a lower degree of fragmentation and a darker color compared to the remaining teas, showing a higher degree of fragmentation and a saturated, light green color. All evaluated samples were characterized by an intense tea aroma and a very bitter taste. No visible lumps were found in the tested products; the powders were loose and dry, with the consistency of a light, aerated powder. No solid impurities were found in the tested products. 

##### Instrumental Measurement of the Color Parameters of Matcha Green Tea Powders

The results of the instrumental color measurement of the tested Matcha samples in the L*a*b* color space are summarized in Table 1. As results from the conducted research, the tested Matcha samples differed significantly (*p* ≤ 0.05) in color parameters. 

In terms of the L* parameter, defining the brightness, significant differences were found in the vast majority of the tested samples. This parameter ranged from 45.90 ± 0.58 to 68.76 ± 2.51, with the highest value in sample M-8 (68.76 ± 2.51) and the lowest in M-10, which means that these products showed the greatest differences in terms of brightness.

The color parameter a*, referring to red (+a*) and green (−a*) in most of the tested samples had negative values, which means that shades of green prevailed in these Matcha samples. The lowest (*p* ≤ 0.05) value of the a* parameter was found in the M-4 (−4.21 ± 0.15), which means that the color of this Matcha tea was shifted towards green shades to the greatest extent. On the other hand, positive values for the a* parameter were found in samples M-3, M-6 and M-10, which means that they were characterized by the largest color shift towards red, with the highest values in M-6 and M-10 (average 0.96 ± 0.04).

In terms of the b* parameter, defining the yellow tone, the highest values were found in samples M-2 and M-7 (average 34.19 ± 0.49), were slightly lower in M-4 and M-8 (average 31.75 ± 0.61) and were the lowest in M-10 and M-6 (average 19.53 ± 0.72), which meant that these products showed the fewest shades of yellow.

##### Water Activity, Water Solubility Index (WSI) and Water Holding Capacity (WHC) of Matcha Green Tea Powders

The results of water activity (*a_w_*), water solubility index (WSI) and water holding capacity (WHC) in the tested Matcha green tea powders are presented in Table 2.

As the conducted research shows, Matcha teas significantly (*p* ≤ 0.05) differed in terms of water activity (*a_w_*), reaching values ranging from 0.1546 ± 0.002 to 0.4082 ± 0.003. The highest water activity (0.4082 ± 0.003) was shown by Matcha M-4 (0.4082 ± 0.003); a lower water activity was shown by M-10 and M-6 (which reached *a_w_* values at the average level 0.3941 ± 0.003), while the lowest *a_w_* (*p* ≤ 0.05) was distinguished by M-9 (0.1546 ± 0.002). All the tested products were characterized by a relatively low water activity, which proves their high durability and microbiological safety, due to the fact that most microorganisms do not develop and grow at a water activity level of *a_w_* < 0.60. 

According to the conducted tests, the water solubility index (WSI) of the tested Matcha powders was 22.09 ± 2.59%, with significant differences in this parameter for the tested products. The highest WSI was found in M-4 and M-1 (average 25.63 ± 0.43%); it was lower in Matcha M-7, M-3, M-8 and M-9, with an average WSI of 22.56 ± 1.07%. The lowest WSI was found in M-6, which dissolved only to about 17% in water at room temperature.

In the case of water holding capacity (WHC), the tested Matcha samples bound an average of 2.50 ± 0.51 g of water per g of powder, with significant differences between the products tested in the study. The highest WHC was shown by Matcha M-6 and M-10 (3.64 ± 0.19 g/g on average); a significantly lower WHC was shown by Matcha M-3 (3.06 ± 0.18 g/g) and M-9 and M-5 (on average 2.45 ± 0.14 g/g). Four of the tested Matcha products, i.e., M-7, M-1, M-2 and M-8, did not differ statistically in this parameter and bound an average of 2.07 ± 0.15 g of water per one gram of powder, while the lowest WHC was in M-4 (1.48 ± 0.09 g/g).

##### pH, Soluble Solids Content and Osmolality in 2.5% Aqueous Solutions of Matcha Green Tea

In the tested market products of Matcha, basic physicochemical parameters such as pH, soluble solids content (°Brix) and osmolality were assessed, and the results are presented in Table 3.

The average pH of aqueous solutions (2.5%) of all tested Matcha teas was 5.71 ± 0.13, with the products significantly (*p* ≤ 0.05) differing in this parameter. The highest pH was found in M-4 (5.94 ± 0.03); it was significantly lower in M-1 and M-2 (average 5.84 ± 0.01) and M-8 and M-7, with an average pH value of 5.74 ± 0.04. The lowest (*p* ≤ 0.05) pH values were found in M-6, M-5, M-9 and M-10 teas (average 5.60 ± 0.04), with no significant differences between these Matcha teas. The average soluble solids content (°Brix) in 2.5% water solutions of Matcha green tea powders was 1.24 ± 0.11%, with the highest ºBrix in the aqueous solution of M-4 tea (1.40 ± 0.10%) and the lowest in M-3 and M-10 (average 1.13 ± 0.05%). As in the case of soluble solids content, the tested Matcha water solutions were characterized by very low osmolality (on average for all samples 2.57 ± 1.77 mOsm/kg·H_2_O), similar to the osmolality of water, although significant (*p* ≤ 0.05) differences in this parameter were found. The lowest osmolality was found in M-9, M-1, M-3, M-6 and M-10, with an average value of 1.20 ± 0.56 mOsm/kg·H_2_O; it was slightly higher in M-2, M-8 and M-5 (2.89 ± 0.78 mOsm/kg·H_2_O), while the highest was shown by two water solutions of the tested Matcha teas, i.e., M-7 and M-4 (5.50 ± 0.55 mOsm/kg·H_2_O).

#### 2.1.2. Bioactive Properties of Matcha Green Tea

##### Phenolic Ingredients in Matcha Green Tea 

**Figure 2:**
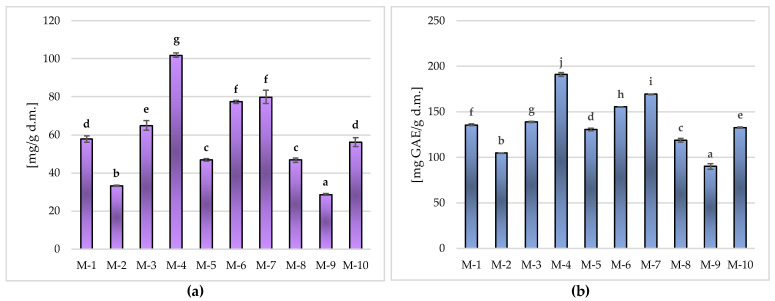
Phenolic ingredients in the tested Matcha green tea samples: the sum of identified phenolic compounds determined by the HPLC method (**a**) and the total polyphenols content measured by the spectrophotometric method (**b**). Mean values marked in bars by different letters (^a–j^) differ significantly (Duncan’s test, *p* ≤ 0.05).

The research carried out in the study showed a high content of phenolic compounds, both determined by the HPLC method (Table 4, Figure 2a) and by the spectrophotometric method using the Folin–Ciocalteu reagent (Figure 2b), in all tested Matcha green teas.

According to the conducted research (Table 4), Matcha green tea was characterized by varied contents of determined phenolic acids, i.e., from 2.96 ± 0.03 to 25.10 ± 0.47 mg/g d.m. (gallic acid) and from 5.78 ± 0.17 to 41.16 ± 0.33 mg/g d.m. (*p*-coumaric acid), with the significantly (*p* ≤ 0.05) highest content of these bioactive ingredients found in M-4 (total 66.26 ± 0.79 mg/g d.m.) and the lowest in the M-2 sample (total 8.74 ± 0.17 mg/g d.m.).

The tested Matcha green teas were characterized by a varied content of flavonoids, and the sum of these compounds ranged from 17.84 ± 0.17 mg/g d.m. (M-9) up to 48.40 ± 0.36 mg/g d.m. (M-7). Among the determined flavonoids, flavanols dominated, including catechins, and the main flavanol in all tested Matcha green teas was gallate epigallocatechin, with an average content of 24.71 ± 8.78 mg/g d.m. The highest (*p* ≤ 0.05) content of this flavonoid was found in samples M-7 and M-6 (average 37.62 ± 2.48 mg/g d.m.), and the lowest was found in M-9 and M-2 (average 11.02 ± 0, 68 mg/g d.m.). The remaining flavanols were present in much lower concentrations, with the epigallocatechin content being over six times lower and the catechin content over eleven times lower, for all analyzed Matcha green tea samples, reaching an average value of 3.99 ± 1.92 mg/g d.m. and 2.20 ± 1.31 mg/g d.m., respectively. 

Regarding the flavanols determined in the tested Matcha samples, the content of rutoside-3-*O*-quercetin was over eight times higher compared to the content of quercetin; the significantly (*p* ≤ 0.05) highest concentration of these flavonoids was found in the M-3 sample (13.00 ± 3.08 and 0.95 ± 0.01 mg/g d.m., respectively), and the lowest (*p* ≤ 0.05) was found in the M-7 sample (2.23 ± 0.34 and 0.23 ± 0.01 mg/g d.m., respectively) (Table 4). Figure 2a shows the sum of identified phenolic compounds determined by the HPLC method. The average content of phenolic bioactive ingredients identified by the chromatographic method was 59.33 ± 21.74 mg/g d.m., and there were statistically significant differences between the tested Matcha green tea samples. The highest (*p* ≤ 0.05) content of these ingredients was found in M-4 (101.91 ± 1.12 mg/g d.m.); it was significantly (*p* ≤ 0.05) lower in samples M-7 and M-6 (average 78.60 ± 2.62 mg/g d.m.), M-3 (64.99 ± 2.44 mg/g d.m.) or M-1 and M-10 (average 56.92 ± 2.08 mg/g d.m.), while the lowest (*p* ≤ 0.05) was found in M-9 (28.62 ± 0.50 mg/g d.m.).

Similar trends were also noted for the content of total polyphenolic compounds, determined by the spectrophotometric method using the Folin–Ciocalteu reagent (Figure 2b).

As can be seen from the data in Figure 2b, the samples were characterized by an average content of total polyphenols at the level of 136.76 ± 28.47 mg GAE/g d.m., with significant (*p* ≤ 0.05) differences between individual Matcha products. The highest polyphenol content was found in Matcha M-4 (190.96 ± 2.14 mg GAE/g d.m.); it was lower in M-7 (169.42 ± 0.43 mg GAE/g d.m.) and M-6 (155.28 ± 0.51 mg GAE/g d.m.). The lowest content of total polyphenols among all tested Matcha samples was recorded in M-9 (89.91 ± 3.17 mg GAE/g d.m.) and it was more than two times lower than in Matcha M-4, with the highest concentration of these bioactive ingredients.

##### Vitamin C and Chlorophylls in Matcha Green Tea

**Figure 3:**
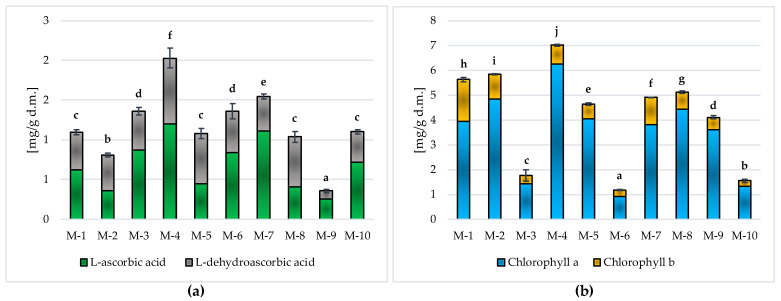
Vitamin C (**a**) and chlorophylls (**b**) content in the tested Matcha green tea samples. Mean values marked in bars by different letters (^a–j^) differ significantly (Duncan’s test, *p* ≤ 0.05).

As can be seen from the data presented in Figure 3a, the tested samples of powdered Matcha green tea differed significantly (*p* ≤ 0.05) in terms of vitamin C content. The highest (*p* ≤ 0.05) concentration of this ingredient was found in sample M-4 (2.03 ± 0.12 mg/g d.m.); it was significantly (*p* ≤ 0.05) lower in M-7 (1.54 ± 0.03 mg/g d.m.), M-3 and M-6 (average 1.36 ± 0.07 mg/g d.m.), M-10, M-1, M-5 and M-8 (average 1.08 ± 0.05 mg/g d.m.) or in sample M-2 (0.81 ± 0.02 mg/g d.m.). The lowest (*p* ≤ 0.05) vitamin C concentration was recorded in sample M-9, in which the amount of this bioactive ingredient was almost six times lower than in sample M-4, reaching only 0.36 ± 0.02 mg/g s.m. 

The average content of total chlorophylls in the tested Matcha green tea samples (Figure 3b) was within a very wide range, i.e., from 1.16 ± 0.03 mg/g d.m. (M-6) up to 7.01 ± 0.05 mg/g d.m. (M-4), and all tested market products differed significantly (*p* ≤ 0.05) in terms of the content of these bioactive ingredients. In all tested Matcha green tea samples, the main chlorophyll was chlorophyll a, whose share was on average 82.68 ± 5.59%.

##### Antioxidant Activity in Matcha Green Tea

**Figure 4:**
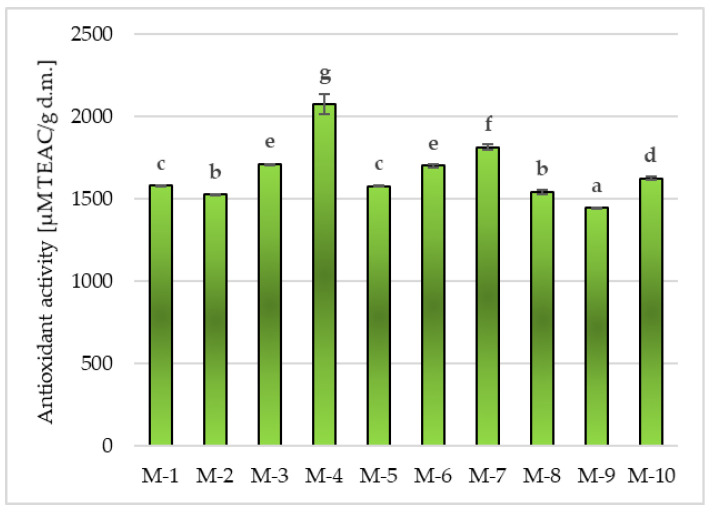
Antioxidant activity in the tested Matcha green tea samples. Mean values marked in bars by different letters (^a–g^) differ significantly (Duncan’s test, *p* ≤ 0.05).

All teas tested in the study were characterized by high antioxidant activity in a range from 1443.56 ± 3.61 µM TEAC/g d.m. (in M-9) to 2076.35 ± 59.11 µM TEAC/g d.m. (in M-4) (Figure 4), with significant (*p* ≤ 0.05) differences between the tested products. Slightly lower antioxidant potential compared to M-4 was found in M-7 (1814.88 ± 14.45 µM TEAC/g d.m.), M-3 and M-6 (average 1705.39 ± 8.11 µM TEAC/g d.m.) or M-10, for which the antioxidant activity reached the values of 1624.94 ± 7.82 µM TEAC/g d.m. Among the Matcha samples tested in the study, M-1 and M-5 showed significantly (*p* ≤ 0.05) lower abilities to deactivate synthetic cation radicals ABTS^+•^, with an average value of 1579.00 ± 4.63 µM TEAC/g d.m., or M-2 and M-8 (1533.53 ± 11.65 µM TEAC/g d.m.).

### 2.2. The Possibility of Using Matcha Green Tea in the Design of Functional Protein Drinks

In the second stage of the research, it was decided to design a model functional beverage with the addition of Matcha green tea. For this purpose, a sample M-4 tea was selected. All tested samples can be a valuable source of polyphenolic compounds in the daily diet, but sample M-4 had the highest antioxidant properties and the highest polyphenol content. Taking into account the obtained results of physicochemical properties, it can be assumed that for the design of a model functional beverage, it would be possible to use any of the tested samples and obtain beverages with similar properties.

The recipe for the composition of two drinks was developed in the form of a ready-to-use powdered mix, which should be mixed with water, milk or another selected liquid product, e.g., a vegetable drink. The recipe composition and appearance of the designed beverages are presented in Table 5.

In both drinks, the addition of maltodextrin as a source of complex carbohydrates and inulin as a source of dietary fiber was used. The addition of inulin and maltodextrin resulted in a slight thickening of the drinks. The drinks also contained the addition of protein preparations—hydrolyzed collagen protein and/or whey isolate. Collagen after dissolving is transparent and tasteless and does not affect the color and taste of the drink; therefore, its addition was used in both drinks. In the case of a drink prepared on the basis of milk, the addition of whey isolate was included in its composition, which also did not affect the color of the drink. The addition of collagen enriched the drink with protein without affecting the taste, consistency and color, while the addition of whey isolate deepened the milky taste of the drink. A slight sediment was visible in the beverages due to the presence of insoluble tea powder fractions.

The first designed drink was to resemble the so-called “Matcha Latte”, which is tea with an addition of milk. To prepare it, milk and tea brewed or mixed with cold water were mixed. One of the advantages of this drink was its characteristic, vivid green color. Matcha is so intense in taste that a small amount is enough to give the drink the right taste. The drink gained a taste characteristic of tea, green, slightly earthy and bitter, balanced by the sweet taste of milk and the addition of sugar.

The second drink designed was a water-based drink. This drink was supposed to be similar to the so-called “Cold Brew Matcha” or “Cold Matcha Tea”, which is mixed with cold water and optionally sweetened as desired.

#### 2.2.1. Physicochemical Evaluation of Designed Functional Protein Drinks

The designed mixes of ready-to-use powders for the drinks preparation and the prepared Matcha functional drinks were evaluated in terms of basic physicochemical characteristics, including *a_w_* (powder mix) and pH, °Brix and osmolality (liquid beverages, ready to drink), and the obtained results are presented in Table 6.

The water activity (*a_w_*) of the ready-to-use powders was 0.186 for the milk-based drink and 0.272 for the water-based Matcha drink. The beverages had pH values ranging from 6.46 for the milk-based drink to 6.58 for the water-based drink. The designed drinks also differed significantly in terms of soluble solids content and osmolality, with significantly (*p* ≤ 0.05) higher °Brix and osmolality for the Matcha drink based on milk (16.33 ± 1.03% and 313 ± 5.65 mOsm/kg·H_2_O). In the Matcha drink based on water, these parameters were about two times lower, 8.83 ± 1.24% (°Brix) and 147 ± 1.63 mOsm/kg·H_2_O (osmolality).

#### 2.2.2. Sensory Evaluation of Designed Functional Protein Drinks

The designed Matcha functional drinks were assessed using the descriptive and point method, paying attention to their features such as their appearance, smell, taste, consistency and overall assessment, and the results of the assessment are presented in Table 7. 

Matcha functional drinks differed depending on the selected liquid component (milk vs. water). The aroma of the drinks, described as herbal, tea, vegetable or sweet, was at a similar level in both variants. Both beverages had a fluid consistency, smooth, free from lumps and large particles, with a marked astringent sensation on the tongue. The taste of the drinks was tea, herbal, slightly sweet and slightly bitter and milky (in the case of a milk-based drink). The overall scores, being a harmonization of all assessed attributes, were high for both designed Matcha drinks. However, it is worth emphasizing that the higher score, i.e., 4.8/5 points, was scored by a milk-based Matcha drink compared to a water-based Matcha drink, which scored 4.0/5 points.

#### 2.2.3. Nutritional Value of Designed Functional Protein Drinks

Table 8 lists the nutritional value of the designed Matcha functional drinks. The energy value of the drinks was about 33–63 kcal/100 mL. The Matcha drink prepared on the basis of water was characterized by a lower energy value; it did not contain fat and salt, and it was characterized by a lower content of protein, sugar and fiber. This was mainly due to the preparation of this water-based drink, without using a lot of ingredients (Table 5). The addition of milk caused a significant increase in energy value, fat, carbohydrate and protein content. Nevertheless, these were not high values. Taking into account the nutritional value of drinks, they can be labeled with nutrition claims in accordance with Regulation (EC) 1924/2006 [38]. The list of claims for individual beverages is also given in Table 8.

## 3. Discussion

### 3.1. Physicochemical and Bioactive Properties of Matcha Green Tea

The conducted organoleptic evaluation of Matcha green tea powders concerning general appearance, degree of fragmentation, granulation, flowability, as well as color, aroma and taste allowed us to conclude that the tested market products were of good quality. In the organoleptic assessment, powdered Matcha green teas were characterized by an intense green color, which proved the correct course of the technological process of its production and the preservation of a large amount of chlorophyll giving the green color [39]. In the case of M-6 and M-10 Matcha samples, which showed a much lower degree of fragmentation (Figure 1), these products were characterized by a less intense color, reminiscent of the typical color of green leaf tea, which made them less attractive than the others. All the tested Matcha samples had an intense tea aroma and were characterized by a distinctly bitter aftertaste, resulting, among others, from the presence of tannins [11].

One of the most important attributes of the appearance of food products, which strongly influences consumer acceptance, is color, and undesirable colors may lead consumers to reject products [40,41]. Considering that the basic selection criterion for tea consumers is precisely this attribute [42], and from the point of view of organoleptic assessment and overall quality of products, it is important to determine the color. A detailed analysis of the color of powdered Matcha green teas with the instrumental method is carried out in this paper and significant (*p* ≤ 0.05) differences between the tested products for individual L*a*b* chromatic coordinates in the CIE color space are demonstrated (Table 1). 

The available literature lacks studies on the instrumental measurement of the color of powdered Matcha green teas. The intense, green color of the tested products resulted from the high content of pigments, mainly chlorophylls [23]. Chlorophylls a and b, which occur in all photosynthetic plants, are among the most common in nature, and their significant content is responsible for the green color of plants [23]. According to the literature, the content of chlorophylls and their derivatives in the leaves of Tencha tea, intended for the production of Matcha, is significantly higher (5.65 mg/g d.m.) than in traditional green tea (4.33 mg/g d.m.) [12]. The research carried out in this study was consistent with the literature data [12]. The results obtained for the total chlorophyll content in the tested samples of powdered Matcha green tea ranged from 1.16 ± 0.03 mg/g d.m. to 7.01 ± 0.05 mg/g d.m. and indicated a high content of these pigments in the 10 tested Matcha products.

**Figure 5:**
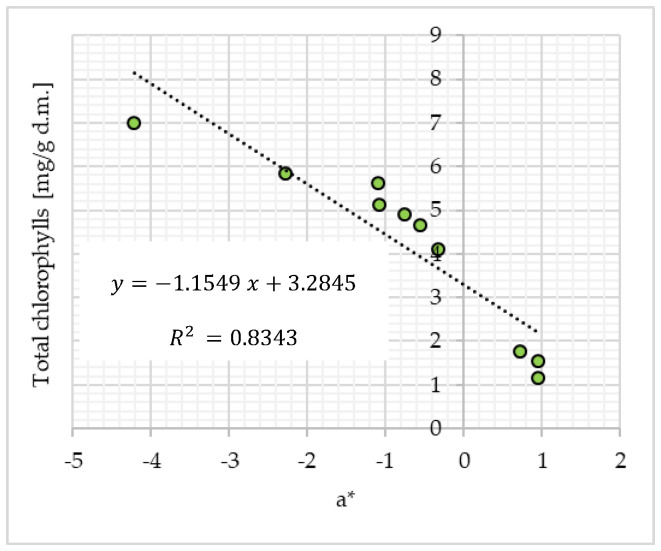
Relation between the total chlorophylls (mg/g d.m.) content and the chromatic coordinate a* (CIE L*a*b*) in the tested Matcha green tea samples.

Taking into account the instrumental color measurement (CIE L*a*b*) (Table 1) and the obtained results of high total chlorophyll content (Figure 3b) in the tested Matcha samples, a significant (*p* ≤ 0.05) relationship between the content of total chlorophylls and the chromatic coordinate a*, which determined the intensity of the green color (−a*) in the tested Matcha green tea powders, was found. This relationship is presented in Figure 5.

The high content of bioactive ingredients, including specific dyes in Matcha green tea, is largely influenced by the technological processes used during its production [11]. Black teas are the result of the complete fermentation of tea leaves, leading to the typical brown-black color, resulting from the enzymatic oxidation of the catechins present in the tea leaves. In green tea production, the phenol oxidase (catalyzing oxidation reactions during the leaf fermentation stage) is deactivated by high-temperature treatment, which allows the green color to be preserved [11]. Differences in the obtained shades of color in the conducted research could result not only from the different impact of technological processes used to obtain powdered Matcha tea [11,16] but also from the degree of fragmentation of the product [26] or agro-climatic conditions of tea cultivation, such as the number and distribution of sunny and rainy days, possible fertilization and plant protection measures during the growth period [25].

The powdered Matcha teas tested in this study were characterized by low water activity (*a_w_*). Many physical, chemical and microbiological changes can occur in food products, and the *a_w_* values can affect the speed and intensity of these changes [43]. Each food product has a specific water activity that affects the adsorption or desorption of water from the environment. Powdered products have a low water activity of 0.15–0.40, e.g., in granulated tea, it is about 0.35 [44]. The average value of this parameter for all products tested in the study was 0.32 ± 0.08, which proves the high durability and microbiological safety of the products, due to the fact that most microorganisms do not develop at water activity at the level of *a_w_* < 0.60 [43].

According to the conducted research, 2.5% water solutions of the tested Matcha teas showed an acid reaction (pH < 7), which also increases the stability of the tested powders by inhibiting the development of microorganisms (e.g., bacteria or yeast) and protection against rotting during storage [43]. In addition, the tested market products had a stable color and intense taste, and, according to the literature, the acidity of teas affects the stability of dyes and enhances the flavor and aroma characteristics [43]. The soluble solids content (°Brix) and osmolality in 2.5% aqueous solutions of Matcha green tea were very low. The content of soluble solids (°Brix) indicates, among others, the content of carbohydrates in the tested solutions, and compared to sweetened beverages, tea has a lower content, which makes it suitable for diabetics or people on a slimming diet [45,46]. The low osmolality of the tested teas allows them to be classified as hypotonic beverages, such as spring or mineral waters [46]. Such drinks are very well absorbed into the body’s cells and can quickly quench thirst [45].

Matcha is characterized by a high content of antioxidant compounds, in particular polyphenols [12,15,24,25,26,27], including, e.g., flavonoids [2,13,16,17,19,21], phenolic acids [13,17,18], carotenoids [12,23] or vitamins, thanks to which it has a strong antioxidant potential [13,14,15,16,17,18,26]. The conducted research showed a high content of phenolic compounds, determined by both the HPLC (Table 4, Figure 2a) and the spectrophotometric (Figure 2b) methods, in tested Matcha green tea samples.

Chromatographic analysis of selected phenolic compounds in the tested Matcha green tea samples (Table 4) showed the dominant share of catechins in the identified flavonoids, which was confirmed by other authors [1,30,31]. According to the literature, the dominant catechins in green tea are epicatechin, epicatechin-3-gallate, epigallocatechin and epigallocatechin-3-gallate [1], with the highest concentration of epigallocatechin-3-gallate [30,31]. The research results obtained in this study confirmed the tendency described in the literature, because gallate epigallocatechin was present in the highest concentration, while the content of epigallocatechin and catechin was over six times and over eleven times lower, respectively, in the tested Matcha green tea samples (Table 4).

The studies of Matcha green tea showed a high content of total polyphenols, ranging for the tested Matcha market products from 89.91 ± 3.17 to 190.96 ± 2.14 mg GAE/g d.m. (Figure 2b), which was confirmed by the test results of other authors. In the studies of Koláčková et al. (2019) [17], the total content of polyphenols ranged from 64.4 to 93.9 mg GAE/g d.m. in aqueous solutions and from 169 to 273 mg GAE/g d.m. in 80% methanol solutions. Analyzing the research results obtained in this work and the other authors results, it can be seen that methanolic solutions (usually at a concentration of 80%) showed a higher content of total polyphenols, which may be justified by a more effective extraction process of polyphenols from tea [17].

In the study by Jakubczyk et al. (2020) [13], the content of total polyphenols in infusions of two types of Matcha green tea was determined: traditional Matcha (from the first and second harvest) and daily Matcha (from the second and third harvest). The content of polyphenols in both infusions was high, but it differed depending on the temperature of the water used to prepare the infusion. The content of flavonoids was also determined in the same study. Infusions were characterized by a high content of these compounds, but higher concentrations were noted in teas from the second and third harvests. The results for traditional Matcha ranged from 1222.60 to 1514.28 mg/L, and for daily Matcha, they ranged from 1379.82 to 1968.79 mg/L.

In other studies [26], it was shown that powdered teas compared to leaf teas were characterized by a higher content of polyphenols using the same amount of leaves and powder during extraction, which may suggest that the grinding process may also affect the increasing the efficiency of extraction of polyphenolic compounds from tea [16,47].

**Figure 6:**
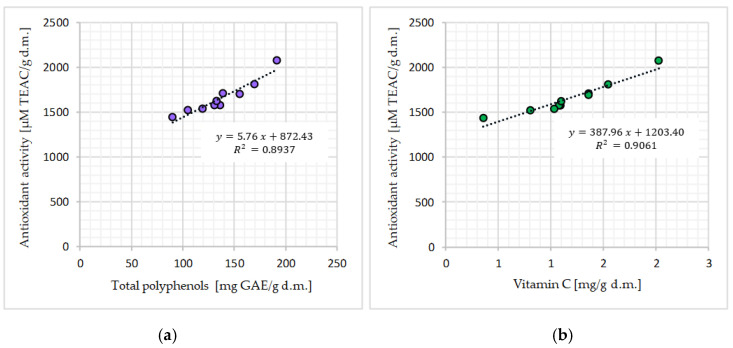
Relation between the total polyphenols (mg GAE/g d.m.) (**a**) or vitamin C content (mg/g d.m.) (**b**) and antioxidant activity (µM TEAC/g d.m.) in the tested Matcha green tea samples.

Based on the research on the content of total polyphenols and the analysis of the literature, it can be concluded that Matcha contains large amounts of bioactive substances, in particular those with antioxidant activity [1]. According to the research, Matcha green teas available on the Polish market were characterized by a high antioxidant potential, ranging from 1443.56 ± 3.61 to 2076.35 ± 59.11 µM TEAC/g d.m. (Figure 4). The obtained results were confirmed in the available literature [17,18,22]. In the studies of Koláčková et al. (2020) [17], the antioxidant activity in Matcha teas from different producers was determined using ABTS^+•^. The authors showed differences in the Matcha antioxidant activity in 80% methanol extracts (from 306 to 368 mg TE/g) and in aqueous extracts (from 246 to 382 mg TE/g). The highest antioxidant activity was found in Matcha methanol extracts. In other studies conducted by Jakubczyk et al. (2020) [13], the antioxidant potential of the tested Matcha tea infusions ranged from 5767.30 to 6129.53 µM Fe^2+^/L. In these studies, higher values were noted in teas brewed at higher temperatures (90 °C), which was most likely related to better release of biologically active compounds and higher kinetic energy at higher temperatures [13]. In other studies on various green teas, for all the analyzed products, an increase in antioxidant potential was shown with increasing water temperature during brewing, and it was found that the highest antioxidant activity was shown by infusions brewed at 100 °C for 3 min [27]. In addition, these authors showed that the antioxidant potential correlates with the content of polyphenolic compounds in the samples they studied, which was also found in the studies obtained in this work (Figure 6a).

The conducted research showed the existence of a significant (*p* ≤ 0.05), positive relationship between the content of total polyphenols and antioxidant activity (R^2^ = 0.8937), as well as between the content of vitamin C and antioxidant activity (R^2^ = 0.9061), measured by the ability to deactivate synthetic ABTS^+•^ cation radicals, as presented in Figure 6. This study is consistent with the results achieved by other authors, who confirmed the high antioxidant properties of Matcha green tea, depending on the high concentration of phenolic compounds and the high vitamin C content [13,16,25,26,47].

In the literature studies, the content of bioactive components and antioxidant activity in Matcha fall within very wide ranges of values. These differences can be explained by the influence of various factors, determining not only the content and profile of phenolic components but also the antioxidant potential in the raw material itself. These factors include species and type of tea [2,3], production processes [13,17,25] and agro-climatic conditions of cultivation [1,25], including the degree of shading and insolation of the plant during its growth [8,12,36] or the conditions of tea harvesting [13,36]. The process of green tea and Matcha production also plays a very important role [25,26]: the form of the final product (leaves, bags, powder) [16,24,26], degree of fragmentation (milling) [26,47] or packaging and storage conditions [48]. The key role in shaping the bioactive properties of consumed products is also played by the methods and conditions of preparing Matcha drinks, including brewing time and temperature [24,47,49].

Due to the richness of bioactive ingredients (mainly polyphenols), especially those with antioxidant properties, Matcha green tea is increasingly used in the prevention and treatment of many diseases, including inflammatory diseases [30,31], viral diseases [20,32] and many civilization diseases, including diabetes [22,50,51], obesity [28], cancer [2], hypertension and heart disease [1,13,22,24,29,52], or for slowing down the aging process [33]

In addition, thanks to its health-promoting properties, as well as specific physicochemical properties (finely ground powder with an intense green color intended for consumption both “hot” and “cold”), Matcha is more and more often used as an addition to various products, including bars, jellies, pastries, drinks, cocktails or ice cream [4,34], becoming a promising ingredient in the functional food industry [22,35,36,37].

### 3.2. The Possibility of Using Matcha Green Tea in the Design of Functional Protein Drinks

The above-presented results of the conducted research on the assessment of physicochemical and bioactive features of various types of Matcha green teas available on the Polish market were the basis for the selection of a product with the most appropriate features for the design of functional beverages. Based on the obtained results, a sample of Matcha M-4 green tea, characterized by the most desirable bioactive properties, was selected for further (design) research. This sample was used to develop the composition of the model beverages that can be recommended to a wide range of consumers, depending on their individual expectations or needs. Thanks to the high content of protein in the drinks, in an easily accessible form, an important group of recipients may be athletes or people with an increased need for protein. Moreover, the addition of maltodextrin, which is an easily available source of carbohydrates, indicates that the drinks can be especially recommended to athletes or physically active people. Maltodextrin is quickly released in the body, thanks to which muscle glycogen stores are quickly replenished and energy is supplied. This ingredient is often used in food products for athletes [53]. It has been shown that the addition of protein to a carbohydrate drink results in better glycogen storage after exercise compared to drinks of the same energy value consisting of carbohydrates or carbohydrates and fat [54]. For the elderly or malnourished, these drinks can also be a helpful supplement to the diet due to the high protein content with low energy value [55]. In addition, the fiber in the form of inulin added to the recipe increases the nutritional value of the drink and is a source of prebiotics. Inulin has prebiotic properties, it can contribute to lowering the concentration of triglycerides and cholesterol in the blood, as well as improving the absorption of minerals such as calcium and magnesium [56]. These ingredients may be present in the liquid component with which the beverage is prepared and thus better absorbed, e.g., calcium in milk or magnesium in water. The bioactive compounds from Matcha present in the recipe composition increase the health-promoting qualities of the prepared drinks.

The available literature contains conflicting data on the effect of adding milk to tea on the antioxidant activity of the beverage so obtained. The differences in the obtained results are mainly due to the use of different types of tea and milk for the preparation of beverages, the composition of the beverage, and the method of their preparation. Interactions between polyphenolic compounds present in tea and proteins present in milk (mainly catechins and caseins) can lead to a decrease in the antioxidant activity of the prepared beverage. Few data on the explanation of this phenomenon can be found in the literature [57]. In addition, milk contains fat globules, which may also interact with tea catechins. Although the negative effect of milk addition on antioxidant activity in tea has been widely described, there is a lack of information on the effect of milk addition on the activity or bioavailability of caffeine in tea infusions [58]. In this study, in the context of sensory characteristics, it was confirmed that the addition of milk to tea infusion improves the taste characteristics of the prepared drink by counteracting the occurrence of astringent and bitter taste. These flavors are characteristic of tea infusions, which is due to the presence of tannins in them. Additionally, the presence of milk in the tea infusion increases the nutritional value of this drink.

Despite the rich and growing market of functional drinks, there is still little information and research on their formulation and design [59]. There is also little research on the interaction of polyphenols with compounds present in food, i.e., carbohydrates, lipids or proteins [60,61,62]. Therefore, the presented research may be the starting point for the design of functional drinks using protein preparations with the addition of powdered Matcha green tea and, in the future, also for testing the bioavailability of polyphenolic components contained in various food products and food matrices.

## 4. Materials and Methods

### 4.1. Materials

The research material consisted of 10 powdered Matcha green teas, which were market products and the most frequently chosen brands in Poland. The products were purchased in specialist tea shops in 100g individual packages. The research was carried out in two stages.

In the first stage of the research, the physicochemical and bioactive properties of 10 selected Matcha products were assessed. An organoleptic evaluation was carried out, the physical properties of samples in the form of powders and 2.5% aqueous solutions of Matcha were assessed, and the bioactive properties were also tested. On the basis of the research results obtained in the first stage, one of ten tested products was selected; it was characterized by the most desirable organoleptic, physicochemical and bioactive properties, which was used to design the composition of functional drinks with the Matcha addition.

In the second stage of the research, the composition of the ready-to-use powdered mix was designed, containing the addition of Matcha green tea as a source of bioactive substances and shaping the taste profile of the functional drinks designed later. The composition was designed, and 2 types of Matcha functional drinks were obtained, i.e., milk-based and water-based. The detailed composition of the beverage formulations is presented in Section 3. Results (Table 5).

### 4.2. Methods

In Matcha powder samples, organoleptic evaluation, measurements of water activity (*a_w_*), instrumental color measurements (L*a*b*), water solubility index (WSI) and water holding capacity (WHC) as well as total polyphenol content and antioxidant activity of the tested 10 market products of Matcha green tea were determined.

#### 4.2.1. Instrumental Color Measurement (CIE L*a*b*) of Powders

Color parameters of Matcha green tea powders were measured at room temperature (20 °C) using a colorimeter (Konica Minolta CR-400, Konica Minolta, BSP, Warsaw, Poland) in the CIE Lab color space (L*a*b*) (L-brightness; +*a*-red; −*a*-green; +*b*-yellow; −*b*-blue). After calibration using a white CR-A43 reflective plate and placing the samples in a glass dish (Ø 60 mm) on the measuring head, the results were read.

#### 4.2.2. Water Activity (*a_w_*) of Powders

Water activity (*a_w_*) of Matcha green tea powders and designed ready-to-use drink powdered mixes were measured using a handheld AquaLab Water Activity Meter (Decagon Devices. Inc., Pullman, WA, USA) with a temperature stabilizer

#### 4.2.3. Water Solubility Index (WSI) of Powders

The water solubility index (WSI) was determined by the gravimetric method according to Yousf et al. (2017) [63]. In plastic Falcone tubes (with a capacity of 50 mL), 2.5 g (with an accuracy of 0.001 g) of Matcha green tea powders were weighed on an analytical balance (AS 220/X, Radwag, Radom, Poland); 30 mL of distilled water was added, mixed in a centrifuge tube, incubated in a heated shaker (IKA KS 4000i Control, IKA^®^ Ltd., Warsaw, Poland) (37 °C ± 1 °C, 30 min) and then centrifuged (MPW-380 R, MPW Med. Instruments, Warsaw, Poland) for 20 min at 10,000 rpm. The supernatant was collected into pre-weighed weighing bottles and dried in an incubator (SUP 200W, Wamed, Warsaw, Poland) at 103 °C ± 2 °C. After drying, it was weighed and the WSI was expressed as % dissolved substance in water.

#### 4.2.4. Water Holding Capacity (WHC) of Powders

The water holding capacity (WHC) was determined by the gravimetric method according to the methodology described by Sudha et al. (2007) [64]. In plastic Falcone tubes (with a capacity of 50 mL), 1.0 g (with an accuracy of 0.001 g) of Matcha green tea powders were weighed on an analytical balance (AS 220/X, Radwag, Radom, Poland); 50 mL of distilled water was added, mixed in a centrifuge tube and then centrifuged (MPW-380 R, MPW Med. Instruments, Warsaw, Poland) for 15 min at 10,000 rpm. Excess water was poured off, and the water-absorbed powder was reweighed and the WHC was expressed as g/g (grams of water per gram of powder).

#### 4.2.5. pH

The pH of 2.5% aqueous Matcha green tea solutions and designed functional drinks was measured by the potentiometric method using a laboratory pH-meter probe (Elmetron CP-511, Elmetron Rp., Zabrze, Poland) at room temperature (20 °C).

#### 4.2.6. Soluble Solids Content (°Brix)

The soluble solids content (°Brix) in 2.5% aqueous Matcha green tea solutions and designed functional drinks was measured using an Abbe refractometer (ORT-1, Kern & Sohn GmbH, Balingren-Frommern, Balingen, Germany), using the refractometric method, according to the Polish Standard (PN-EN 12143:2000) [65]. The results (expressed in %) were read from the sugar scale at room temperature (20 °C).

#### 4.2.7. Osmolality

The osmolality of 2.5% aqueous Matcha green tea solutions and designed functional drinks was measured using an osmometer (Osmometr Krioskop 800CL, Trident Med. Clp, Warsaw, Poland) by measuring the crystallization temperature of a supercooled solution with a one-point calibration. After measuring 100.0 μL of the sample into the osmometric tube and placing it in the cooling chamber, as a result of supercooling the sample and initiating crystallization, the heat of crystallization was measured with a thermistor and automatically converted to mOsm/kg·H_2_O.

#### 4.2.8. Selected Phenolic Acids and Flavonoids Identified by the HPLC Method

In plastic sterile test tubes (10 mL capacity), 0.1 g (accuracy to 0.001 g) of powdered Matcha green tea was weighed on an analytical balance (AS 220/X, Radwag, Radom, Poland); 5.0 mL of 80% methanol (Sigma-Aldrich, Poznań, Poland) was added and vortexed (Wizard Advanced IR Vortex Mixer, VELP Scientifica Srl, Usmate, Italy) (30 s, 2000 rpm) to mix thoroughly, and then incubated in an ultrasonic bath (PolSonic, Warsaw, Poland) for 10 min (5.5 kHz, 30 °C). After the incubation, the samples were centrifuged in a refrigerated centrifuge (MPW-380 R, MPW Med. Instruments, Poland, Warsaw) for 15 min (6000 rpm, 2 °C), and the obtained supernatants (1.0 mL) were collected into the chromatographic vials and analyzed by HPLC. 

The contents of selected phenolic acids and flavonoids in Matcha green tea were determined by the HPLC method, according to Hallmann et al. (2017) [66], using the Shimadzu HPLC kit (USA Manufacturing Inc., Waltham, MA, USA), consisting of two LC-20AD pumps, a CMB-20A system controller, a CTD-20AC controller, a SIL-20AC autosampler and a UV/VIS SPD-20AV detector. Identification and separation of selected phenolic compounds were performed on a Synergi Fusion-RP 80i chromatographic column (250 × 4.60 mm) (Phenomenex, Shimpol, Warsaw, Poland), using a flow gradient with two phases: acetonitrile/deionized water (55% and 10%) at pH 3.00. The analysis time was 38 min, with a flow rate of 1.0 mL/min and detection at λ = 250–370 nm. External standards, i.e., gallic acid, *p*-coumaric acid, catechin, epigallocatechin, gallate epigallocatechin, quercetin and rutoside-3-*O*-quercetin, with a purity of 99.98% (Fluka and Sigma-Aldrich Poznań, Poland), were used to identify the substances. Five injections of phenolic standard solutions were made in order to prepare standard curves. The content of selected phenolic compounds was calculated based on the standard curves, and the results were expressed as mg/g d.m. (d.m.—dry matter).

#### 4.2.9. Vitamin C Content

In plastic sterile test tubes (10 mL capacity), 0.1 g (accuracy to 0.001 g) of powdered Matcha green tea was weighed on an analytical balance (AS 220/X, Radwag, Radom, Poland); 5.0 mL of 5% metha-phosphoric acid (Sigma-Aldrich, Poznań, Poland) was added and vortexed (Wizard Advanced IR Vortex Mixer, VELP Scientifica Srl, Usmate, Italy) (30 s, 2000 rpm) to mix thoroughly, and then incubated in an ultrasonic bath (PolSonic, Warsaw, Poland) for 10 min (5.5 kHz, 30 °C). After the incubation, the samples were centrifuged in a refrigerated centrifuge (MPW-380 R, MPW Med. Instruments, Poland, Warsaw) for 10 min (6000 rpm, 0 °C), and the obtained supernatants (1.0 mL) were collected into the chromatographic vials and analyzed by HPLC. 

The vitamin C content in Matcha green tea was determined by the HPLC method, according to Ponder and Hallmann (2020) [67], using the Shimadzu HPLC kit (USA Manufacturing Inc., Waltham, MA, USA), consisting of two LC-20AD pumps, a CMB-20A system controller, a SIL-20AC autosampler and a UV/VIS SPD-20AV detector. Identification and separation of L-ascorbic and L-dehydroascorbic acids were performed on a Hydro 80-A RP column (250 × 4.6 mm) (Phenomenex, Shimpol, Warsaw, Poland), using a mobile phase: 0.1 M acetic acid (glacial, 99.9% purity) (Sigma-Aldrich, Poznań, Poland) and 0.1 M sodium acetate (Sigma-Aldrich, Poznań, Poland) (in volume proportions of 63:37 *v*/*v*) at pH 4.4. The analysis time was 18 min, with an isocratic flow of 1.0 mL/min and detection at λ = 255–260 nm. External standards, i.e., L-ascorbic acid (L-ASC) and L-dehydroascorbic acid (L-DHA) (Sigma-Aldrich, Poznań, Poland), with a purity of 99.5%, were used to identify the substances. Five injections of L-ASC and L-DHA standards solutions were made in order to prepare standard curves. The vitamin C content was calculated based on standard curves, and the results were expressed (as the sum of L-ASC and L-DHA acids) in mg/g d.m. (d.m.—dry matter). 

#### 4.2.10. Chlorophylls Content

In plastic sterile test tubes (10 mL capacity), 0.1 g (accuracy to 0.001 g) of powdered Matcha green tea was weighed on an analytical balance (AS 220/X, Rad-wag, Radom, Poland); 5.0 mL of cold acetone (Sigma-Aldrich, Poznań, Poland) and 10.0 mg of magnesium carbonate MgCO_3_ (Sigma-Aldrich, Poznań, Poland) were added and vortexed (Wizard Advanced IR Vortex Mixer, VELP Scientifica Srl, Usmate, Italy) (30 s, 2000 rpm) to mix thoroughly, and then incubated in an ultrasonic bath (PolSonic, Warsaw, Poland) for 10 min (5.5 kHz, 0 °C). After the incubation, the samples were centrifuged in a refrigerated centrifuge (MPW-380 R, MPW Med. Instruments, Poland, Warsaw) for 10 min (6000 rpm, 0 °C), and the obtained supernatants (1.0 mL) were collected into the chromatographic vials and analyzed by HPLC.

The chlorophyll content in Matcha green tea was determined by the HPLC method, according to Hallmann et al. (2017) [66], using the Shimadzu HPLC kit (USA Manufacturing Inc., Waltham, MA, USA), consisting of two LC-20AD pumps, a CMB-20A system controller, a SIL-20AC autosampler and a UV/VIS SPD-20AV detector. Identification and separation of chlorophyll a and chlorophyll b were performed on a Synergi Max-RP 80A column (250 mm × 4.60 mm) (Phenomenex, Shimpol, Warsaw, Poland), using two mobile phases: acetonitrile/methanol (Sigma-Aldrich, Poznań) (90:10 *v*/*v*) and methanol/ethyl acetate (Sigma-Aldrich, Poznań) (64:36 *v*/*v*). The analysis time was 28 min, with a flow rate of 1.0 mL/min and detection at λ = 450 nm. External standards, i.e., chlorophyll a and chlorophyll b, with a purity of 99.98% (Sigma-Aldrich Poznań, Poland) were used to identify the substances. Five injections of chlorophyll a and chlorophyll b standard solutions were made in order to prepare standard curves. The total chlorophyll content was calculated based on the standard curves, and the results were expressed (as the sum of chlorophyll a and b) in mg/g d.m. (d.m.—dry matter).

#### 4.2.11. Preparation of Water Extracts for Total Polyphenol Content and Antioxidant Activity Assay

In plastic sterile Falcone tubes with a cap (50 mL capacity), 0.5 g (accuracy to 0.001 g) of powdered Matcha green tea was weighed on an analytical balance (AS 220/X, Radwag, Radom, Poland); 40 mL of distilled water was added and vortexed (Wizard Advanced IR Vortex Mixer, VELP Scientifica Srl, Usmate, Italy) (60 s, 2000 rpm) to mix thoroughly, and then incubated in a shaking incubator (IKA KS 4000i Control, IKA^®^ Ltd., Warsaw, Poland) for 60 min (60 °C, 200 rpm). After incubation, the samples were again vortexed for 60 s for thorough mixing and then centrifuged in a refrigerated centrifuge (MPW-380 R, MPW Med. Instruments, Warsaw, Poland) for 15 min (4 °C, 10,000 rpm). The supernatant obtained in this way was used to determine the total polyphenol content and antioxidant activity in Matcha green tea.

#### 4.2.12. Total Polyphenol Content 

Total polyphenol content in Matcha green tea was determined using the Folin–Ciocalteu reagent (Sigma-Aldrich, Poznań, Poland) according to the Singleton and Rossi (1965) [68] method. The solution (1.0 mL of diluted supernatant, 2.5 mL of Folin–Ciocalteu reagent, 5.0 mL of 20% Na_2_CO_3_ (sodium carbonate) in 41.5 mL of distilled water) was incubated for 60 min (20 °C, no access to light) and the absorbance was measured in a spectrophotometer (UV/Vis UV-6100A, Metash Instruments Co., Ltd., Shanghai, China) at λ = 750 nm. After taking into account the dilutions used, the obtained results of absorbance measurements were recalculated based on the standard curve (y = 2.127x + 0.1314, R^2^ = 0.9994) for gallic acid (Sigma-Aldrich, Poznań, Poland) as a standard substance and the total polyphenol content was expressed as mg GAE/g d.m. (GAE—gallic acid equivalent, d.m.—dry matter).

#### 4.2.13. Antioxidant Activity 

Antioxidant activity in Matcha green tea was determined using the cation radical ABTS^+•^ (2,2‣-azino-bis 3-ethylbenzothiazolin-6-sulfonic acid) (Sigma-Aldrich, Poznań, Poland) according to the Re et al. (1999) [69] method. The solution (1.5 mL of diluted supernatant, 3.0 mL radical cations ABTS^+•^ in PBS solution (PBS—phosphate buffer solution)) was vortexed (Wizard Advanced IR Vortex Mixer, VELP Scientifica Srl, Usmate, Italy) and incubated for 6 min (20 °C), and the absorbance was measured in a spectrophotometer (UV/Vis UV-6100A, Metash Instruments Co., Ltd., Shanghai, China) at λ = 734 nm. After taking into account the dilutions used, the obtained results of absorbance measurements were recalculated based on the standard curve (y = –5.6067x + 0.7139, R^2^ = 0.9998) for Trolox (Sigma-Aldrich, Poznań, Poland) as a standard substance and the antioxidant activity was expressed as µM TEAC/g d.m. (TEAC—Trolox equivalent antioxidant capacity, dm.—dry matter).

#### 4.2.14. Sensory Evaluation of Functional Matcha Drinks

Sensory evaluation of the designed functional Matcha drinks was carried out by six panelists with extensive experience in assessing the sensory characteristics of various products using the descriptive and point method. The drinks were assessed in the following categories: taste, smell, color, consistency and overall rating on a five-point scale (5—very good, 4—good, 3—sufficient, 2—insufficient, 1—bad).

#### 4.2.15. Nutritional Value of Functional Matcha Drinks

The nutritional value of the designed functional Matcha drinks was calculated on the basis of the information provided on the labels of the beverage components regarding their energy value and the content of individual nutrients and salt. The study calculated the energy value, fat content (including saturated fatty acids), carbohydrate content (including sugars), fiber, protein and salt content of the prepared drinks.

#### 4.2.16. Statistical Analysis

The differences between the samples were considered statistically significant at *p* ≤ 0.05. Statistical analysis of the obtained results was performed using the Statistica 13.0 software (Tibco Software Inc., Palo Alto, CA, USA). The significance of differences between the obtained results was determined by performing a one-way analysis of variance (ANOVA). In order to show the differences between the individual groups, the Duncan test was used at the assumed significance level of *p* ≤ 0.05. The results were presented in tables and graphs in which the division into homogeneous groups was marked and the mean values and standard deviations (SD) were presented. 

## 5. Conclusions

The Matcha green teas tested in the study, in the organoleptic assessment regarding the general appearance, degree of fragmentation, granulation, flowability, as well as color, aroma and taste, were characterized by good quality and their solubility in water, giving mostly homogeneous mixtures with a pleasant, typical tea aroma and intense green color. The soluble solids content of 2.5% aqueous solutions of Matcha green tea powders was close to the °Brix of pure water, and their osmolality was within the range of hypotonic beverages. Knowledge of such properties is crucial due to the possibility of using Matcha green tea powder as a source of bioactive ingredients in the design of functional food and various types of food products with its participation. 

The tests carried out showed a high content of bioactive ingredients, including selected phenolic acids and flavonoids identified by the HPLC method, as well as total polyphenols, determined by the spectrophotometric method using the Folin–Ciocalteu reagent. Moreover, the tested Matcha green tea samples were characterized by a high content of chlorophylls, vitamin C and high antioxidant activity.

Functional food is a constantly developing market segment. It affects health and can prevent the development of civilization diseases. Many active or nutritional ingredients are used in its production. Currently, most products are enriched with proteins, pro and prebiotics, polyunsaturated fatty acids and antioxidants, including polyphenols. Beverages are the most extensive category of functional foods. Despite the great interest in the group of functional products, which include the designed drinks, there is little research on their formulation, the interaction of the ingredients of such food as well as the sensory characteristics of protein drinks with the addition of various protein preparations and additional inulin preparation. Due to the wide range of health-promoting properties of Matcha green tea, it is worth including this product in daily diet, both in the form of functional drinks or as an addition to other food products, such as cocktails, desserts or snacks. In the present study, a formulation of an innovative ready-to-drink protein drink was developed, containing both Matcha bioactive ingredients and digestible whey proteins, amino acids and peptides of enzymatically hydrolyzed collagen proteins rich in bone-joint tissue hydroxyamino acids (hydroxyproline and hydroxylysine) and the prebiotic inulin. This preparation, when dissolved in the consumer’s preferred liquid (water, milk, juice), constitutes a ready-to-drink regenerative drink of low osmolality (an iso- and hypotonic drink). This beverage can be recommended to a wide range of consumers, including physically active people and malnourished people with protein deficiency, and as a functional beverage, it can be used in the prevention and treatment of connective tissue diseases.

## Figures and Tables

**Table 1:** Color parameters (CIE L*a*b*) in the tested Matcha green tea powders.

Sample	L*	a*	b*
M-1	63.15 ± 0.58 ^d^	−1.10 ± 0.02 ^c^	30.17 ± 0.74 ^d^
M-2	59.82 ± 0.31 ^c^	−2.29 ± 0.11 ^b^	34.04 ± 0.36 ^g^
M-3	55.25 ± 1.45 ^b^	0.71 ± 0.09 ^g^	26.69 ± 0.58 ^b^
M-4	56.28 ± 2.10 ^b^	−4.21 ± 0.15 ^a^	31.51 ± 0.69 ^ef^
M-5	56.44 ± 0.31 ^b^	−0.55 ± 0.04 ^e^	30.40 ± 1.11 ^de^
M-6	46.35 ± 1.45 ^a^	0.96 ± 0.06 ^h^	19.78 ± 0.73 ^a^
M-7	64.10 ± 0.72 ^d^	−0.74 ± 0.04 ^d^	34.35 ± 0.62 ^g^
M-8	68.76 ± 2.51 ^e^	−1.07 ± 0.05 ^c^	31.99 ± 0.39 ^f^
M-9	57.02 ± 0.35 ^b^	−0.33 ± 0.07 ^f^	28.80 ± 0.60 ^c^
M-10	45.90 ± 0.58 ^a^	0.94 ± 0.04 ^h^	19.28 ± 0.45 ^a^

Mean values ± standard deviation with different letters (^a–h^) in the same column differ significantly (Duncan’s test, *p* ≤ 0.05).

**Table 2:** Water activity (*a_w_*), water solubility index (WSI) and water holding capacity (WHC) in the tested Matcha green tea powders.

Sample	Water Activity (*a_w_*)	WSI (%)	WHC (g/g)
M-1	0.2655 ± 0.001 ^c^	25.45 ± 0.56 ^f^	2.04 ± 0.01 ^b^
M-2	0.3000 ± 0.000 ^d^	22.14 ± 0.48 ^d^	2.04 ± 0.16 ^b^
M-3	0.3024 ± 0.000 ^d^	23.30 ± 0.45 ^e^	3.06 ± 0.18 ^e^
M-4	0.4082 ± 0.003 ^h^	23.33 ± 0.20 ^e^	2.19 ± 0.21 ^bc^
M-5	0.2238 ± 0.001 ^b^	21.05 ± 0.37 ^c^	2.44 ± 0.16 ^cd^
M-6	0.3926 ± 0.003 ^g^	17.43 ± 0.76 ^a^	3.71 ± 0.02 ^f^
M-7	0.3743 ± 0.001 ^f^	25.80 ± 0.22 ^f^	1.48 ± 0.09 ^a^
M-8	0.3375 ± 0.001 ^e^	22.72 ± 0.18 ^de^	2.01 ± 0.14 ^b^
M-9	0.1546 ± 0.002 ^a^	20.89 ± 0.23 ^e^	2.47 ± 0.14 ^d^
M-10	0.3956 ± 0.004 ^g^	18.80 ± 0.21 ^b^	3.56 ± 0.26 ^f^

Mean values ± standard deviation with different letters (^a–h^) in the same column differ significantly (Duncan’s test, *p* ≤ 0.05).

**Table 3:** pH, °Brix and osmolality of 2.5% aqueous Matcha green tea solutions.

Sample	pH	°Brix (%)	Osmolality (mOsm/kg·H_2_O)
M-1	5.84 ± 0.01 ^c^	1.23 ± 0.06 ^abc^	1.00 ± 0.00 ^a^
M-2	5.83 ± 0.02 ^c^	1.30 ± 0.10 ^cde^	2.33 ± 0.58 ^bc^
M-3	5.59 ± 0.02 ^a^	1.13 ± 0.06 ^a^	1.00 ± 0.00 ^a^
M-4	5.94 ± 0.03 ^d^	1.40 ± 0.10 ^e^	5.67 ± 0.58 ^d^
M-5	5.61± 0.02 ^a^	1.27 ± 0.06 ^bcd^	3.33 ± 0.58 ^c^
M-6	5.63 ± 0.09 ^a^	1.17 ± 0.06 ^ab^	1.67 ± 0.58 ^ab^
M-7	5.72 ± 0.05 ^b^	1.37 ± 0.06 ^de^	5.33 ± 0.58 ^d^
M-8	5.75 ± 0.02 ^b^	1.27 ± 0.06 ^bcd^	3.00 ± 0.00 ^c^
M-9	5.61 ± 0.04 ^a^	1.13 ± 0.06 ^a^	0.67 ± 0.58 ^a^
M-10	5.58 ± 0.04 ^a^	1.17 ± 0.06 ^ab^	1.67 ± 0.58 ^ab^

Mean values ± standard deviation with different letters (^a–e^) in the same column differ significantly (Duncan’s test, *p* ≤ 0.05).

**Table 4:** Selected phenolic compounds identified by the HPLC method in the tested Matcha green tea powders.

Sample	Gallic Acid	P-Coumaric Acid	Catechin	Epigallocatechin	Gallate Epigallocatechin	Quercetin	Rutoside-3-*O*-quercetin
M-1	3.75 ± 0.19 ^b^	20.64 ± 0.53 ^f^	3.41 ± 0.10 ^e^	3.43 ± 0.33 ^d^	22.67 ± 1.03 ^b^	0.45 ± 0.01 ^d^	3.46 ± 0.18 ^a^
M-2	2.96 ± 0.03 ^a^	5.78 ± 0.17 ^ab^	2.49 ± 0.15 ^d^	5.51 ± 0.11 ^f^	10.41 ± 0.15 ^a^	0.75 ± 0.02 ^e^	5.22 ± 0.37 ^b^
M-3	2.86 ± 0.06 ^a^	15.94 ± 0.77 ^e^	1.68 ± 0.05 ^c^	4.43 ± 0.21 ^e^	26.13 ± 1.35 ^c^	0.95 ± 0.01 ^g^	13.00 ± 3.08 ^c^
M-4	25.10 ± 0.47 ^h^	41.16 ± 0.33 ^g^	0.80 ± 0.01 ^a^	6.61 ± 0.08 ^g^	25.66 ± 0.70 ^c^	0.29 ± 0.00 ^bc^	2.31 ± 0.34 ^a^
M-5	6.58 ± 0.08 ^c^	7.32 ± 1.31 ^cd^	1.14 ± 0.05 ^b^	1.39 ± 0.24 ^b^	27.39 ± 0.42 ^c^	0.31 ± 0.01 ^c^	2.66 ± 0.13 ^a^
M-6	22.65 ± 0.05 ^f^	6.27 ± 0.37 ^bc^	0.69 ± 0.01 ^a^	6.68 ± 0.03 ^g^	38.45 ± 0.26 ^d^	0.28 ± 0.01 ^b^	2.30 ± 0.12 ^a^
M-7	24.16 ± 0.50 ^g^	7.54 ± 0.61 ^d^	4.70 ± 0.05 ^f^	4.25 ± 0.40 ^e^	36.79 ± 3.63 ^d^	0.23 ± 0.01 ^a^	2.23 ± 0.34 ^a^
M-8	7.33 ± 0.39 ^d^	6.48 ± 0.67 ^bcd^	3.52 ± 0.22 ^e^	1.00 ± 0.02 ^a^	25.49 ± 0.11 ^c^	0.44 ± 0.03 ^d^	2.51 ± 0.09 ^a^
M-9	3.63 ± 0.19 ^b^	7.15 ± 0.43 ^cd^	1.03 ± 0.06 ^b^	2.38 ± 0.09 ^c^	11.62 ± 0.10 ^a^	0.29 ± 0.00 ^bc^	2.52 ± 0.28 ^a^
M-10	18.79 ± 0.46 ^e^	4.95 ± 0.08 ^a^	2.51 ± 0.06 ^d^	4.19 ± 0.12 ^e^	22.46 ± 2.08 ^b^	0.84 ± 0.03 ^f^	2.29 ± 0.07 ^a^

Mean values ± standard deviation with different letters (^a–h^) in the same column differ significantly (Duncan’s test, *p* ≤ 0.05).

**Table 5:** Recipe composition of functional drinks with Matcha flavor based on milk and water.

Component	Matcha Drink Based on Milk		Matcha Drink Based on Water	
General appearance of Matcha drinks	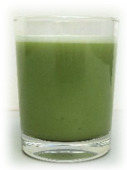		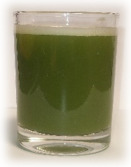	
	[g/100 g drink]	[g/100 g powder]	[g/100 g of drink]	[g/100 g powder]
Water	43	-	90	-
Milk 1.5% fat	43	-	-	-
Inulin	3	21	2.5	25
Collagen hydrolyzate	3	21	3	30
Whey isolate	3.5	25	-	-
Maltodextrin	2.5	18	2.5	25
Sugar	1.5	11	1.5	15
Matcha M-4	0.5	4	0.5	5

**Table 6:** Water activity (powder mix), pH, soluble solids content (°Brix) and osmolality of designed Matcha functional drinks.

Parameter	Matcha Drink Based on Milk	Matcha Drink Based on Water
Water activity (*a_w_*)	0.19 ± 0.00 ^a^	0.27 ± 0.01 ^b^
pH	6.46 ± 0.05 ^a^	6.58 ± 0.02 ^b^
°Brix	16.33 ± 1.03 ^b^	8.83 ± 1.24 ^a^
Osmolality (mOsm/kg·H_2_O)	313.00 ± 5.65 ^b^	147.00 ± 1.63 ^a^

Mean values ± standard deviation with different letters (^a,b^) in the same line differ significantly (Duncan’s test, *p* ≤ 0.05).

**Table 7:** Evaluation of organoleptic features of designed Matcha functional drinks by descriptive and point method.

Sensory Features	Matcha Drink Based on Milk	Matcha Drink Based on Water
Appearance	light green color with a delicate sediment and foam on the surface	dark green color, cloudy drink
Points (0–5)	4.8	4.0
Smell	milk, tea, vegetable, herbal, sweet	herbal, tea, spicy, sweet, vegetable, grassy
Points (0–5)	4.5	4.2
Taste	milk, tea, herbal, slightly sweet, slightly bitter, vegetable	vegetable, tea, herbal, almond, slightly sweet, bitter, burning, astringent, raw, grassy
Points (0–5)	4.8	4.0
Consistency	liquid, causing astringency on the tongue, perceptible fine particles suspended in the drink	fluid, slightly viscous, causing tightening on the tongue
Points (0–5)	4.7	4.0
Overall score (0–5)	4.8	4.0

**Table 8:** Nutritional value of designed functional Matcha drinks in 100 mL of beverage.

Nutritional Value/ Nutrition Claim	Matcha Drink Based on Milk	Matcha Drink Based on Water
Energy value (kJ/kcal)	265.19/63.34	139.29/33.27
Fat (g)	0.7	<0.1
Saturated fatty acids (g)	0.47	<0.01
Carbohydrates (g)	8.8	6.25
Sugars (g)	4.28	2.18
Proteins (g)	6.7	2.7
Fiber (g)	2.7	2.25
Salt (g)	0.05	<0.01
Nutrition claims according to Regulation (EC) 1924/2006	High protein content Low fat Low salt content High content of dietary fiber	High protein content Does not contain fat It does not contain salt High content of dietary fiber

## Data Availability

Not applicable.
